# Husserlian Phenomenology as a Kind of Introspection

**DOI:** 10.3389/fpsyg.2018.00896

**Published:** 2018-06-06

**Authors:** Christopher Gutland

**Affiliations:** Department of Philosophy, Center for Documentation and Research of Phenomenology, Sun Yat-sen University, Guangzhou, China

**Keywords:** Husserl, phenomenology, introspection, consciousness research, methodology, a priori, Kant

## Abstract

The thesis of this article is that Husserl's proposed method for intuitively exploring the essential or *a priori* laws of consciousness is a kind of introspection. After a first reflection on the meaning of “introspection,” four elements of Husserl's methodology are introduced: the principle of all principles, *epoché*, phenomenological reduction, and eidetic variation. These features are then individually related to six common features Eric Schwitzgebel mentions in his definition of introspection in the *Stanford Encyclopedia of Philosophy*. The explanation of these elements is complemented by mentioning phenomenological insights they offer. It is thereby shown how Husserl's methodology evades some of the pitfalls of introspection and reaches a secure ground. Such pitfalls are: a relatively uncontrolled and varying scope of awareness, false prejudices, and problems distinguishing between idiosyncratic and general features of consciousness. As this article is written for the section *Theoretical and Philosophical Psychology*, Husserl's approach is developed in relation to two well-known philosophical systems that considerably influenced him, Hume's and Kant's.

## Introduction

The majority of phenomenologists keep phenomenology and introspection strictly apart (see Thomasson, 2003, p. 239; Smith and Thomasson, 2005, p. 9; Zahavi, 2007, p. 76; Staiti, 2009c, p. 231; Fuchs, 2015, p. 809). While Husserl (1971, p. 38) distanced his method from psychology's inner observation, he occasionally characterized it as introspection (see Husserl, 1973c, p. 23; De Palma, 2015, p. 203). In line with this, more recent thinkers openly employ phenomenology for introspective endeavors (see Shear and Varela, 1999; Depraz et al., 2003)^1^. So what is the relation between phenomenology and introspection?

While Husserl clearly distinguished between phenomenology and psychology, he (see Depraz, 1999, pp. 103–105; Staiti, 2009a) nonetheless mentioned a psychological path into phenomenology. And if one wishes to walk it, this article argues, introspection is the most feasible starting point. Relatedly, Husserl (1977, p. 6) named Wilhelm Dilthey as a pioneer who struggled to discover a method to study “internal experience.” Yet he was dissatisfied with Dilthey's “contrasting of naturalistically externally directed and descriptively internally directed psychology” (Husserl, 1977, p. 10). For the Cartesian dualism underlying the crude internal-external dichotomy misleadingly makes it seem inner experience is one homogenous field of *res cogitans* (see Husserl, 1970, pp. 211–15). Rather, within what one could call “internal experience” (see section Defining Introspection below), there are *multiple layers* in need of clear differentiation, for instance, the psychological, transcendental, and bodily planes.

Above and beyond the heterogeneity of internal experience, Husserl (1977, p. 11) asks: “[I]n a psychology which relies solely upon internal experience and description of psychic life, how do we arrive at universalities of law?” This shows that Husserl was not *per se* against research drawing on inner experience, especially given that he wanted to describe the general laws of consciousness *based on actually experiencing them*. Instead, his concern was how to methodologically distinguish between individual (idiosyncratic) and general aspects *within it*.

Remarkably, many researchers distancing phenomenology from introspection assume that introspection only yields idiosyncratic results peculiar to a certain individual's consciousness. There is, however, no reason to limit introspection's focus to idiosyncratic experiences only. In order to fully grasp and answer Husserl's question of how to distinguish between general and idiosyncratic features of consciousness, it makes sense to assume that introspection as such yields both: *idiosyncratic and general* experiences (see Breyer and Gutland, 2016, pp. 12–14). This also fits Husserl (1983, p. 41) claim that “all human beings see ‘ideas,' ‘essences,' and see them, so to speak, continuously.” The difficulty thus lies in *clearly noticing* these essential structures as such within consciousness.

This article therefore argues that phenomenology is not opposed to introspection *per se*, but is rather an attempt to refine it by making it scientific and systematic. It does so by providing a method for identifying and describing the general features within consciousness. The structure of this article focuses on introducing Husserl's method in an accessible manner. Where fitting or helpful, it also relates Husserl's phenomenology to features commonly associated with introspection and also to the philosophies of Hume and Kant.

## Defining introspection

In his article on introspection in the *Stanford Encyclopedia of Philosophy*, Schwitzgebel (2016) notes: “No simple characterization is widely accepted.” Instead of a unitary definition, he lists features that many introspective accounts have in common. Accordingly, and instead of arbitrarily defining introspection such that it neatly fits Husserl's methodology, the following article relates Husserl's method to these features Schwitzgebel lists.

To gain a first idea of introspection, one can note with Schwitzgebel (2016) that the word derives “from the Latin ‘looking into.”' The word “into” evokes the notion of space, but is used *only metaphorically* to indicate a shift of attention to an experience that has *no place in external space at all*. How can we positively state its direction? One can note that the external world, beyond its mere existence, also *appears to us*. And this *appearance of* the world is an *experience* without spatial location in the external world. In a Husserlian sense, introspection is aimed at this experience and the way of experiencing it.

An example: Suppose you visit the Galleria dell'Accademia in Florence to look at the original of Michelangelo's David. After you enter, David's statue appears for the first time in your field of vision. Yet you are tacitly aware that the statue existed for hundreds of years before. Thus, the beginning of its appearance *in your consciousness* does not coincide with the beginning of *the statue's existence*. As you approach the statue, its appearance increases in size relative to your visual field. When you are close enough, a part of it may even cover your entire visual field. Tacitly, however, you are aware that the appearance's increase in size does not imply an increase in size of the existing statue. Furthermore, as you move around the statue, it appears to you from different angles. Again, tacitly, you know that this shift of angles is due to your movement, and that the statue stands still. Finally, when you leave and see David no more, you are tacitly aware that the statue's existence has not ended, but only its appearing to you.

After reading this description, you probably notice that in your everyday life you rarely pay heed to how the world consciously appears to you. Instead, our interest is in the existing world and the things existing in it. Of course, we are often concerned with appearances—for example, the way we or others look. But this interest, too, is geared at the presence, absence, or arrangement of *existing* things like clothes, hair, accessories, and so on. In contrast, we are only rarely concerned with appearances as phenomena of consciousness^2^. Furthermore, this appearance itself is nowhere externally observable by others like a marble statue is. You may experience other visitors looking at the statue, but you do not experience *their conscious experiences* of the statue. The conscious experience of appearances thus contrasts with external space and everything in it (including processes in our brain). Sensing this oppositeness to what is *externally* observable, one can see why people began speaking of *intro*spection to express the different direction that an observation of consciousness takes. While it is certainly confusing to use a *spatial direction* metaphorically to indicate something *without place in space*, seen this way, the use of “introspection” is at least understandable.

Understanding introspection this way fulfills the first of the six conditions Schwitzgebel (2016) mentions, the so called “*mentality condition*: Introspection is a process that generates, or is aimed at generating, knowledge, judgments, or beliefs about *mental* events, states, or processes, and not about affairs outside one's mind, at least not directly”^3^. The introduction of the *epoché* below will make this shift of focus clearer.

Concordantly the word “introspection,” as used here, refers to the study *not of the external world*, but of the way we are *conscious of* it, as well as other mental phenomena which have no place in external space. The following sections show how Husserl proposed to do this.

## An outline of husserl's phenomenological methodology

Husserl's method is easier to grasp when one sees it as overcoming problems the better-known philosophies of Hume and Kant encountered.

### Husserl's reaction to hume—or, the principle of all principles

Hume (2007, p. 45) famously asked on what basis we assume that within the world there are necessary connections, such as causality. He assumed that “all our ideas are nothing but copies of our impressions.” He consequently suggested that, to investigate ideas like causality, we “[p]roduce the impressions or original sentiments, from which the ideas are copied” (Hume, 2007, p. 46). This leads him to famously claim that only “when […] the same object is always followed by the same event; we then begin to entertain the notion of cause and connexion. We then *feel* […] a customary connexion […] and this sentiment is the original of that idea which we seek for” (Hume, 2007, pp. 56–57). Hume (2007, p. 51) stressed, however, that this feeling is *not* an experience of necessary causality itself.

Husserl accepted Hume's assertion of an intuitive givenness of any theoretical proposition that is to be thought of as necessary (or essential). This is clearly visible from what Husserl (1983, p. 44) calls the “*principle of all principles*: *that every originary presentive intuition is a legitimizing source of cognition, that everything originarily […] offered* to us *in ‘intuition' is to be accepted simply as what it is presented as being*, but also *only within the limits in which it is presented there*. We see indeed that each theory can only again draw its truth itself from orginiary [sic] data.” Thus, the meaning of “intuition” in Husserl is such that it can fulfill as well as falsify our (theoretical) convictions.

Demanding that *all* elements of a theory be given intuitively before it is deemed true is notably distinct from the scientific method. Starting in the nineteenth century, science began using hypothetical-deductive reasoning (see Carrier, 2009, p. 18). Since then, unobserved elements were accepted in scientific theories if they led to predictions confirmed by empirical observation. The consequence was the Duhem-Quine indeterminacy: Two or more theories, distinct through their postulated unobserved elements, could equally well predict the course of actual empirical observations (see Carrier, 2009, p. 20). A famous example of this phenomenon in physics is Bohmian mechanics vs. the standard model of quantum mechanics. Both theories predict the observable events equally well, but they do so by postulating quite different unobserved elements. When multiple theories have equal empirical footing, a common suggestion is to prefer the one requiring fewer hypothetical elements—a practice known as “Ockham's razor.”

As Husserl demands a *strict correlation* between *all* elements of a theoretical proposition and actual observation, he has no need for Ockham's razor. For the principle of all principles forbids hypothetical elements. This requirement is in line with what Schwitzgebel (2016) calls the “*directness condition*: Introspection yields judgments or knowledge about one's own current mental processes relatively *directly* or *immediately*.” Mental experiences are not to be inferred, logically deduced, or hypothesized, but instead, must be *actually experienced* if one wants to claim the existence of an introspective experience.

The principle of all principles, furthermore, fulfills what Schwitzgebel (2016) calls the “*temporal proximity condition*: Introspection is a process that generates knowledge, beliefs, or judgments about one's *currently ongoing* mental life only.” Describing today what I experienced a week ago would be against the principle of all principles, as this principle requires one to describe what *presents itself* in the here and now. Nonetheless, describing acts of remembering is possible, as a memory is something that one experiences in the here and now even though one experiences it in such a way that one sees the current experience as *re-presenting* what *presented* itself at an earlier point in time. Similarly, descriptions of anticipations, expectations, etc. are possible.

Yet the question arises: What does Husserl do with elements like causality? Given Hume's concern, how does Husserl achieve intuitive fulfillment for a categorial relation like causality? This is the point where one has to look at Kant's reaction to Hume in order to better understand Husserl's solution.

### Kant's reaction to hume

Kant saw the danger Hume's skepticism posed for science and rejected an empirical deduction of causality. Instead, he proposed that our experience, long before we make any conscious judgments about it, is already and necessarily *structured* by categories like causality. In a peculiar way, he thereby both agreed and disagreed with Hume. He agreed with Hume in that he assumed concepts like causality are not connected within (analytic) thinking, but instead need a non-conceptual (synthetic) carrier in order to be combined. However, he disagreed with Hume in that he rejected establishing relations such as causality based on actual empirical experience (a posteriori). Instead he suggested that actual experience as we know it is only possible if it is already (a priori) structured by categories like causality. Thus, although experience *is* the synthetic carrier that establishes necessary connections like causality, these connections' necessity cannot be established by observing experience.

But how do categories become experiential structures? In order to explain this, Kant (1999, A 24–25/B 39, A 31–32/B 47) unconventionally proposed seeing space and time not as conceptual relations, like Aristotle (1963, pp. 1b−2a) did, but as both pure intuitions and forms of intuition (see Kant, 1999, A 20/B 34–35). After this reinterpretation, Kant was able to utilize time and space as *non-conceptual carriers* for conceptual relations. He claimed that causality *appears* to us *in the form of a certain temporal succession* and that this succession of perceptions is *necessarily* so, if the events have a causal connection. Thus, a category like causality is proposed as necessarily (a priori) inherent to our experience by means of its dictating a certain temporal succession of appearances.

Kant furthermore used space and time to establish an unbridgeable gap between the world *in itself* and the world *as it appears to us*. He claimed space and time are subjective necessities of the way the world appears to us humans. With the word “subjective,” Kant does not mean the *individual* human subject, but all humans. Space and time are *subjective* relative to the world *in itself*. They are, however, at the same time *objective* relative to the way it *appears to us*, as for us experience of objects is *impossible* without these structures. Space and time are thus necessary filters of human experience. Kant (1999, A 42/B 59) maintained that once we abstract from the way the world appears to us humans, “space and time themselves would disappear.” Therefore, these filters distort the experience of the things and the world as they are in themselves.

This distinction between appearances and things in themselves is notably different from the distinction between the statue of David's *appearance* and its *physical existence* provided above. The description above was such that we get to know the existing statue *by means* of its appearing and without assuming something like an unknowable statue in itself. This is an important difference between Husserl's and Kant's accounts of experience, which will be further elaborated now.

### Some of husserl's reactions to kant

Husserl rejected Kant's distinction between appearances and things in themselves and wanted “to radically deracinate the false transcendence that still plays its part in Kant's 'thing-in-itself' doctrine and to create a world concept that is purely phenomenological” (Husserl, 2008, p. xxxix, my translation). Thus, for him, a physical thing is not an appearance of an incomprehensible thing in itself. Instead, Husserl (1983, p. 92, see also 2003, p. 67, 2004, p. 129) saw it as “fundamentally erroneous to believe that perception […] does not reach the physical thing itself.”

Second, Husserl rejected Kant's route of access to knowledge about a priori structures. Kant (1999, A 35/B 52) stated that “no object can ever be given to us in experience that would not belong under the condition of time.” If, however, all intuitions and experiences we can have are already temporal, we cannot intuitively study how temporality and sense intuition *become interwoven* in the first place. As a result, Kant's access to the processes preceding our experience is *speculative*. Kant's (1999, A 91-92/B 123–124) was well aware of this, as he clearly rejected establishing causality's necessity based on experience (a posteriori). He pointed out that his entire system is ultimately a thought experiment that aims to achieve verification by means of *being thinkable* without contradiction (see Kant, 1999, B xviii–xix).

Husserl (1970, p. 115) took issue with these speculations about intuitively inaccessible processes allegedly shaping our actual experience. He complained that Kant resorted to a “mythical concept formation. He forbids his readers to transpose the results of his regressive procedure into intuitive concepts […]. His transcendental concepts are thus unclear in a quite peculiar way.” Husserl consequently sought to intuitively explore the conscious processes shaping experience as we know it.

One important feature that Husserl (1960, p. 144, 1970, p. 199) did accept was Kant's so called “Copernican turn.” In order to explain how we, as subjects, can have knowledge about objects, Kant (1999, B xvi-xvii) suggested that we conceive of the object's appearance based on forms that we find in ourselves as experiencing subjects. In line with this, Husserl (1960, p. 114) postulated an “‘*innate' Apriori*, without which an ego as such is unthinkable.” This explains why he (see Husserl, 1968, pp. 250, 300, 328, 344) assumed our world experience is relative to an absolute, transcendental subjectivity that constitutes it.

Husserl likewise accepted Kant's (1999, A 51/B 75) claim: “Thoughts without content are empty, intuitions without concepts are blind.” Adopting this means that one *always* needs to look out for the proper *correlation* between any given intuition *and* concept, as only together can they be meaningful. Kant (1999, A 240/B 299) elucidates further: “[I]t is also a requisite for one **to make** an abstract concept **sensible**, i.e., to display the object that corresponds to it in intuition, since without this the concept would remain (as one says) without **sense**.” As the categories are concepts, this transfers to them as well. Thus, in a similar vein, Husserl (2001b, p. 306) wrote: “It lies in the nature of the case that everything categorial ultimately rests upon sensuous intuition, that […] an intellectual insight […] without any foundation of sense, is a piece of nonsense.” Husserl always asked for a sensory foundation when a priori (eidetic) structures are to be explored phenomenologically.

### Interim summary

In summary, the assumptions upon which Husserl's methodological approach rests are,

(a) With Kant, Husserl assumes that there are *a priori laws* governing conscious states and processes.
(b) He furthermore assumes that these laws are enforced through the activity of a transcendental subjectivity.
(c) They are nonetheless *the same for everyone*.
(d) They are also thus *generalizable* results of an introspective exploration of consciousness.
(e) In contrast to Kant and in line with Hume, Husserl strives to explore these laws *based on intuition*.
(f) Finally, he assumes with Kant that concepts and intuitions need to be explored in strict correlation.

Given these assumptions, a number of questions arise that must be addressed in the following sections,

1. If Husserl rejects Kant's “things in themselves,” how does he conceive of and study the relation between the appearance of an object and the object appearing?
2. How does Husserl rule out the possibility that our prejudices and biases distort our descriptions?
3. Which methodological steps does Husserl take in order to achieve reliable grounds for introspective research?
4. Drawing on experience, how does Husserl avoid his results having only, as Kant (1999, B 3) put it, “assumed and comparative **universality** (through induction)”?
5. How does Husserl ensure that his method yields results that are independent from the peculiarities of the individual observer's consciousness?

### The phenomenological *epoché*: entering introspective grounds

In comparing David's statue to its appearance, it was noted that we are naturally preoccupied with the existing world and the objects existing in it. In order to shift awareness toward the way the world appears to us, Husserl (1983, pp. 57–60) advises becoming disinterested in the things' and, ultimately, the entire world's *existence*. The idea is that once you become detached from wondering what something *actually is*, you have the necessary freedom to study *how its appearance is related to what you think it is*. Husserl calls this “the phenomenological *epoché*” (
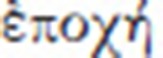
). What thereby happens to our natural convictions about existence is called “bracketing” or “parenthesizing.” Husserl (1983, p. 61) clarifies: “I am *not negating* this ‘world' as though I were a sophist; I am *not doubting its factual being* as though I were a skeptic; rather I am exercising the ‘phenomenological' 
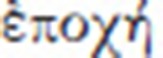
 which also *completely shuts me off from any judgment about spatiotemporal factual being*.”

Husserl (1977, p. 145) comments that the epoché's “*not* having as theme or abandoning from the thematic domain […] is an essential change of the way in which the object-consciousness […] is executed.” Thus it is a *shift* of attention. This is relevant for introspection insofar as there is a crucial distinction between what is happening in consciousness and what we *notice* about it. The Husserlian epoché is a means of *becoming aware* of conscious processes that usually go by unnoticed. Therefore, if you practice epoché, it is not that you genuinely create the aspects of consciousness you become aware of, but rather you shift your attention toward them.

This reveals an important ambiguity of the word “conscious”: It can refer to conscious processes and phenomena that are there regardless of anyone taking *explicit notice* of them, or it can mean *their being noticed*. Therefore, the question whether one is conscious of David's appearance increasing in size when approached can be answered with yes and no. For it is a fact that it does increase, yet usually we do not pay explicit attention to this. In order to establish an unambiguous terminology, from now on the words “conscious” and “consciousness” are used to refer to processes and phenomena independent of their being the focus of attention or not. The words “aware” and “awareness,” on the other hand, are used to highlight the fact that someone becomes aware of a conscious process or state that she did not explicitly notice before^4^.

This terminological contrast allows the identification of a naïveté which is one of the reasons why introspection has “a bad track record” (Spener, 2011, p. 280). A common misconception is that once we introspect, we can readily report *everything* going on in consciousness. That is certainly false, for not only are we usually aware only of certain aspects of consciousness, but it is also questionable whether our awareness can ever encompass the entirety of consciousness. Therefore, an important question for any introspective approach is: How do we achieve awareness of the different aspects of consciousness and how can we be sure we did not miss any (see Smith, 2005, p. 95)?

For Husserl, the answer lies primarily in different gradations of the epoché. We can bracket some or even all discrete objects. A further step is to bracket ourselves as existing human subjects. The final and most encompassing epoché brackets not only all objects and empirical subjects, but the entire world, which is the universal horizon (see Husserl, 1959, p. 161). This bracketing of the entire world, including us as empirical subjects, is at the same time the entry gate into what Husserl called “transcendental phenomenology.”

The epoché as a means to become aware of aspects of consciousness which are always there but usually pass by without us noticing fulfills two of the features Schwitzgebel lists.

One is the “*detection condition*: Introspection involves some sort of *attunement to* or *detection of* a *pre-existing* mental state or event, where the introspective judgment or knowledge is (when all goes well) *causally* but not *ontologically* dependent on the target mental state” (Schwitzgebel, 2016). In other words: The epoché does not genuinely create the existence of what we experience in it. Instead, it lets us become aware of the rich conscious life which is there whether we practice epoché or not^5^.

This is a tricky point, however, as we might wonder about the precise relation between the change of experience implied in *becoming* aware of something and the feature of consciousness that we thereby become aware *of*. Is the experience of awareness *identical* to the feature of consciousness we become aware of, or is there a *distance*? Related is the worry that introspection, attention, and reflection might modify or distort what we hope to experience through them. Regarding this worry, one must first realize that a *change of givenness* is the very *reason why* we employ these techniques: Namely, we wish to experience something better, more clearly, more fine grained, and so on. This change, however, only concerns the *form* of givenness, not the given *content*.

In this context, Husserl (1983, pp. 181–90) furthermore discusses a critique by H. J. Watt, who claimed phenomenology was impossible because the experience we have without reflection is radically distinct from what we observe reflectively or introspectively. Husserl provides two counterarguments: First, he (see Husserl, 1983, p. 185f) emphasizes that Watt himself needs to reflect on his experience of doubt about reflection in order to express it and thereby *uses* what he wishes to render *useless*. Second, to claim a radical modification from pre-reflective to reflective awareness, one needs to be in an adequate position to *compare* the two states, i.e., to have secure knowledge about the pre-reflective awareness. Husserl (1983, p. 186) critically remarks that thus “*knowledge* of reflectionally unmodified mental processes […] is continuously presupposed, while at the same time the possibility of that knowledge is placed in question.” Therefore, claiming a distinction between pre-reflective and reflective experience begs the question of how the implied positive and definite awareness of the pre-reflective is achieved^6^.

To be sure: Reflection is not the only means to become aware of something conscious. It is beyond the scope of this article to provide a detailed comparison of reflection and attention. Yet it is important to note: Drawing on Husserl, Zahavi (2015, p. 186) claims that “attention is a particular feature or mode of our primary act,” whereas “reflection is a new (founded) act.” Breyer (2011, pp. 249 and 252) furthermore suggests seeing reflection as a radicalization of attentive awareness in order to spot meaningful structures. In this article, “to notice” and “to be aware of” encompass both: attentional directedness and reflective awareness^7^.

The other feature is the “*effort condition*: Introspection is not *constant, effortless, and automatic*. We are not every minute of the day introspecting. Introspection involves some sort of special reflection on one's own mental life that differs from the ordinary un-self-reflective flow of thought and action” (Schwitzgebel, 2016). Accordingly, one can see the epoché (also the reduction discussed below) as “un-natural […] in the sense that it is contrary to the natural attitude” (Depraz et al., 2003, p. 99). Experience of the transcendental sphere thus requires the epoché as both: an unnatural effort and a means of detection.

### Immanent object, noema, and thing

When you practice epoché, you may notice that you experience David three-dimensionally. However, at any given moment, only one of David's many sides appears to you^8^. Wherever you move, there will always be an invisible rear side, as well as other distances from which you can experience David. If you have seen them previously, you will have a more definite and detailed expectation of what they look like. But even then, an *actual* impression of the current rear side is missing. Unbeknownst to you, a malevolent visitor could have applied red paint to the rear side, so your memory of it would differ from its current state.

This possible difference between your expectation of the statue's rear side and its actual rear side is indicative of a further difference. In consciousness, you obviously have a way to both remember and anticipate features of David—and yet the actual David can differ. The way you learn about the actual David being different from your expectation is via the actually appearing side. Therefore, three “components” are involved in perceiving David: (1) the currently appearing side, (2) what you think David is like, and (3) the actual David, to which you have a “live feed” through the currently appearing side.

In principle, what you think David to be like can be in full correspondence with the actual David. Experiencing David from *all* sides and distances *simultaneously*, however, is impossible. You can only experience one side as actually given, and this side never encompasses everything there is to experience about David in that moment^9^. In this sense, a *complete* experience of David is impossible, while a partial one is possible. The impossibility of experiencing a thing from all sides is one reason why Husserl (1999, p. 27) said that a thing always *transcends* (exceeds) what is given of it in an actual experience^10^.

However, you can also focus your awareness on the appearance in such a way that you disregard the transcendent aspects just described. From this perspective, you do not experience the phenomenon *as a side* of some *thing*, but as a pure “this-here” (Husserl, 1999, p. 24) in your visual field. This result of looking at the “bare phenomenon” is what Husserl calls the “immanent object.” The immanent object, at least at first glance, is not missing anything and is therefore “*an absolute givenness*” (Husserl, 1999, p. 24). This absolute givenness of the immanent object contrasts with the way the transcendent object is never given with all its features.

Yet, importantly, Husserl (1999, pp. 27–28) discovered a second meaning of “transcendent.” The first meaning refers to the fact that a thing always has features *beyond* the ones currently given to you in experience. The second meaning can be illustrated with Aristotle's (2016, p. 65) claim that “the stone is not in the soul, but rather its form.” When you perceive a stone, your perception *is not* the stone, but it is a *perception of* a stone. For instance, unlike the actual stone, you cannot pick up your perception of the stone and throw it into a pond. Nonetheless, your perception of the stone is a *form* in which you can be *consciously aware of* a physical object like a stone and even its physical properties (like density and shape). Still, the stone remains a physical object whether you perceive it or not. This means that you can be *conscious of features* which are not in themselves *features of consciousness*. Now, Husserl also uses the word “transcendent” to refer to such objects and their features that are not themselves of consciousness. The stone *as a physical thing* is hence a “transcendent object” in this second sense.

Both meanings of “transcendent” refer to something that is in a sense *outside of consciousness*. The first meaning refers to the fact that the actual experience of a thing always *lacks impressions* of it. These absent impressions, however, *can in principle become conscious* (though not all simultaneously). The second meaning refers to features *of which* we *are conscious*, but which themselves *are not features of consciousness*. Mechanical causality, for instance, is applicable to a stone, but not to the *perception of* the stone. It is thus necessary to distinguish between the “perception of a stone” as a conscious entity and the “actual stone” as a physical entity, which is transcendent in the second sense^11^.

Husserl calls the conscious object we experience when we perceive transcendent objects like a stone the “noema”^12^. He chooses a tree to illustrate this. The tree as the transcendent object or the “physical thing belonging to nature […] can burn up, be resolved into its chemical elements” (Husserl, 1983, p. 216). The tree as the noema “cannot burn up; it has no chemical elements, no forces, no real properties” (Husserl, 1983, p. 216).

The two meanings of “transcendent” imply that there are two meanings of “immanent” as well. The sense of “immanent” already introduced refers to the fact that at a given moment in time *only some* of the features of an object *give themselves in experience*. However, *in a way*, these absent features *are nonetheless experienced*, even if they *do not give themselves* in experience. For *you assume* the object *to have* these features even though they are absent as far as current impressions are concerned. You can, for instance, become aware of the color *you* think David's rear side has and describe it as *you* expect it to be. In fact, without any such assumptions about the absent aspects of a physical object, it could not be experienced at all. For if you turn an object, you become aware of *new* aspects of the *same* object, but these aspects need a conception of the object that anticipates them or at least leaves room for them.

You may have noticed that this second meaning of immanent is thus identical to the noema. For the noema—*the way you are conscious of* a thing that is *not itself of consciousness*—is through and through a conscious phenomenon which can be described as such. This means that the noema is *transcendent* relative to the immanent object, and at the same time *immanent* with regard to the thing which is itself not of consciousness. In phenomenology, it is as difficult as it is crucial to make these distinctions. Husserl (1983, p. 308) himself admits that his *Logical Investigations* still mostly lack descriptions of the noema. To express the peculiar status of the noema, one could also call it a “transcenden*tal*” rather than “transcend*ent*” object, as it refers to the way we are *consciously aware* of something *transcendent to consciousness*^13^.

In order to avoid ambiguous terminology, from here on, in this text:

(1) “**Immanent object**” refers to the “bare” conscious phenomenon without all the intentions of currently absent impressions.
(2) “**Noema**” or “**transcenden*tal* object**” refers to phenomena which are:
  (a) *transcendent* in the sense of *including* all the intentions abstracted from in order to be aware of the immanent object; and
  (b) still *immanent* to consciousness in contrast with “consciousness-external” things.
(3) “**Thing**” or “**transcend*ent* object**” refers to that which is not itself of consciousness (like the stone as a physical object) and hence is transcendent in the second sense.

These distinctions, particularly the one between noema and thing, are very important for psychology and introspection alike. Without them, there is a confusing and dangerous ambiguity to the word “object” (see Kaiser-el-Safti, 2015, p. 5). This seminal distinction is an important starting point for understanding how, for example, *something physical* can be *experienced consciously*. If this were not possible, it would be hard to conceive how sciences like physics and chemistry could have emerged at all.

In order to contrast the scientific approach and the phenomenological approach, one can say: A scientist studies the properties (e.g., physical, chemical) of transcendent objects and pays no heed to the way she is conscious of them by means of noemata. A phenomenologist, on the other hand, studies the properties of the noemata by becoming disinterested in the properties of transcendent objects.

This only contains a grain of truth, however, as within the history of science it became rather dubious whether experiential qualities like colors or odors are objective properties at all. Galilei (1957, p. 274), Newton (1952, pp. 124–25), and others claimed that these experiential qualities are entirely subjective. Husserl (1970, pp. 23–59) pointed out that science only accepted as objective those experiential qualities, like geometrical shape, that allowed for a *direct quantification*. Because of this, science studies *only certain features* of our experience of things, while it dismisses the others as “merely subjective.” This leads to claims that these experiential qualities could be reduced to natural processes altogether. Against this, Frank Jackson (1986) wrote his famous article “What Mary didn't know.” I can only hint here, however, at the possibility of reconsidering the status of the so-called secondary sense qualities (or qualia) based on a phenomenological analysis.

The effect of this opinion on qualia is that science is looking for “the real world” as something *distinct* from the way we experience it. This is akin to Kant's claim that our experience of objects is distinct from the objects in themselves^14^. As a consequence of the assumption that we do not experience the world as it is, we need to assume that our experience *does not present* the world to us, but instead *represents* it in some *indirect* fashion. Husserl firmly disagreed with this assumption for reasons the next subsection addresses.

### Perception does not represent an object, it presents it

Because he refutes Kant's speculations about things in themselves, for Husserl there is no David in itself which is forever beyond the reach our conscious experience. For the noema allows you to be conscious of David's statue even with its physical properties. So there is no need to look for the “real David” beyond the way you are conscious of it. Instead, you can be conscious of David *as it is* (though only partially in the first sense of “transcendent”).

Following Husserl (2001b, p. 284), perception “gives the object ‘presence' in a simple, immediate way.” Thus it does not *re*-present things, it *presents* them. We can, however, also represent a thing. We do so, for instance, when we remember or imagine an object. In such cases, it is *us*, with our mental activity, who *re-*present an object. In contrast to this, a thing *presents itself* in perception. Acts like remembering or expecting remain relative to this way of presentation, which is why they *re*-present. Also, if you *imagine* a green cactus, you can choose to recolor it magenta. But if you *perceive* a green cactus, no similar mental action or intention yields a color change. While perceiving, as Shields (2011, p. 232) puts it, the subject's “will is impotent in the face of the phenomenal.” This is indicative of perception offering an *originary* contact with an object, as here the object *presents something of itself* without any distortion of the subjective will^15^.

Both science's and Kant's assumption that the “real world” is distinct from the way we experience it are potent contemporary prejudices preventing us from seeing that things *present themselves* in perception. Kant (1999, A 320/B 376) claimed that “[t]he genus” of all conscious phenomena “is **representation** in general.” Many other philosophers followed, notably Schopenhauer (2010, p. 23), who claimed: “‘The world is my representation': – this holds true for every living, cognitive being.” Husserl (2001a, p. 276) disagrees and goes so far as to say that understanding all conscious experiences as representations (*Vorstellungen*) “is one of the worst conceptual distortions known to philosophy. It is without doubt responsible for an untold legion of epistemological and psychological errors.”

It is here where the contrast between Husserl and Kant becomes revealing regarding introspection. If introspection means to study the way the world *appears to us subjectively* in consciousness, the problem arises that in Kant's philosophy the way the world appears to us *subjectively* already entails all we will ever know about the world *objectively*. For the categories of the understanding are woven into our subjective experience by means of its spatiotemporal structure. These are the same structures we explicate in conscious judgments about the world. Beyond that, we will never know how the world is *in itself*. Introspection, in Kant, would thus peculiarly yield both subjective and objective knowledge. Husserl instead rejects speculations about an unknowable world in itself and sees the world itself as being *consciously presentable* in the form of noemata, even where the presented aspect of the world is not itself of consciousness. Yet the possibility of nonetheless distinguishing between the noema and the transcendent object allows us to draw a clear line between phenomenology and sciences like physics. This is notably absent in Kant, which is why introspection in his system would fail to provide exclusively subjective results.

Husserl (1983, p. 92) claims that those who seek the “real” world beyond the one we experience are “misled by thinking that the transcendence belonging to the spatial physical thing is the transcendence belonging to something *depicted* or *represented by a sign*.” We can of course be conscious of something by means of a sign, but Husserl (1983, p. 93) maintains “an unbridgeable essential difference” between this signitive consciousness and perception. For when “we intuit something in consciousness as depicting or signitively indicating something else; having the one in our field of intuition we are directed, not to it, but to the other” (Husserl, 1983, p. 93). Instead, in “immediately intuitive acts we intuit an ‘it itself;' […] there is no consciousness of anything *for which* the intuited might function as a ‘sign' or ‘picture”' (Husserl, 1983, p. 93). This is why, for Husserl (1983, p. 92), it is “fundamentally erroneous to believe that perception […] does not reach the physical thing itself,” and that is also his answer to question (1) raised above.

The result of this section, expressed in Aristotelian terminology, is: The fact that we experience a thing in the *form* of being *conscious of it* does *not* preclude us from knowing its non-conscious *features (contents)*. We can therefore perfectly well be *conscious of* something transcending consciousness, for example a law of classical mechanics, which is not a law *of consciousness*. Even science presupposes this. Otherwise, it would be hard to explain how someone reading a book on physics thereby extends her knowledge about physical laws rather than laws of consciousness. And the physical is only one of many transcendent layers which are accessible by means of being conscious of them.

The following sections focus on the noema and mostly ignore transcendent objects. This means, however, entering a world very much unknown to us in our everyday lives. In order to find *orientation* in it, another methodological technique is required.

### The phenomenological reduction^16^: adjusting introspection

The epoché is “the gate of entry” (Husserl, 1970, p. 257) to a sphere that remains unknown to us in everyday life. But we bring something with us: our knowledge and experience. As the sphere we enter is largely unknown, however, our habitual assumptions and judgments that are true for the transcendent world might turn out to be dangerous prejudices here. What Husserl therefore demands is similar to a general amnesia of prejudices or a state of presuppositionlessness, as Zahavi (2003, p. 44) calls it.

The suppression of prejudices, which Husserl calls for, is only one methodological requirement for achieving a truthful description, however. There is another that seems to escape Husserl's attention. For not only do we need to *withhold* the blind application of concepts that we are *already familiar* with (i.e., prejudices) we also need to *acquire new concepts* in order to be able to accurately describe the new sphere. In other words: We need not only to watch and describe what we see without prejudice, as Husserl (1956, p. 147) makes it seem, we need to *learn*. This learning is not only one of new words or new word usage, it is also the acquisition of new meanings. The association of these meanings with the words used in describing is a further and also problematic step (see Smith, 2005, p. 99) which is addressed in more detail in Critical Discussion of Husserl's Method. One reason why Husserl overlooked the acquisition of meanings genuinely new to the describing subject was that he (see 1960, p. 114) assumed an a priori innate to the ego. Assuming this rules out not only the necessity, but also the possibility, of acquiring new meanings. For they are all innate, ready to be spontaneously applied whenever called for. A second reason why Husserl overlooked this requirement is that he (see Husserl, 2001b, pp. 260–261) assumed meanings were in need of a sensory intuition in order to be given adequately. This assumption renders it superfluous to ask for conceptual content *in addition* to a sensory intuition, as the latter seemingly provides the concept's meaning.

These two prejudices of Husserl are important for understanding his proposed methodology. For the answer to the question of how to make sure that a proposition truthfully describes the newly entered sphere is simply: It has to be in full accord with what we intuitively experience. This process is thus a *reduction* of our description to exactly what we experience and is thereby Husserl's answer to questions (2) and (3) raised above.

While this may sound simple, it is in fact one of the most challenging methodological requirements. Overcoming our prejudices as blind mechanisms of judging, which normally happen to us passively and without notice, is an arduous task. The worry, however, is not that prejudices are always wrong. They can be right or wrong with regard to a given experience. It is their *blind application*, their *passive* happening to us, that is dangerous. For if they are wrong and we do not notice them, they distort our attempts to accurately describe our experience.

To conclude: Husserl (1977, p. 67) believed that the effort to align a description strictly to what is experienced can be successful and, if so, yield a usage of concepts with an adequate intuitive basis. Yet even if one grants that such a description can adequately express an experience, how do we ensure that the experience is generalizable and not idiosyncratic to a particular individual's consciousness? In order to show how Husserl's method overcomes this obstacle, the concept of intentionality and the act of imagining need to be introduced.

### Intentionality, noesis, and motivation

While the noema always lacks intuitive givenness of *all* its features, it is nonetheless entirely introspectively accessible and discernible (see Husserl, 1970, pp. 241–243). For instance, I can easily notice and introspectively describe *what I assume* David to be like. While the result may sound like a description of a transcendent object, it is nonetheless a description of the way we are *conscious of* David. Husserl (1983, p. 216) emphasizes that “these descriptive statements, even though they may sound like statements about actuality, have undergone a *radical* modification of sense.” That is: We are no longer concerned whether David's rear side *really is* textured like we assume, but we pay attention to the fact that we assume him to have a certain rear side and begin to wonder *how and why* we do that.

The reason the noema is fully accessible to introspective inquiry is that it is a strict correlate of a *conscious act*, which is also introspectively accessible. Husserl calls this act the “noesis.” Both these words derive from the ancient Greek ν*oε*ĩν—“to think” or “to understand.” When we *perceive* something, however, the noesis has a presupposition: the immanent object. Therefore, leaving out the transcendent object, when we perceive there are three elements involved: (1) the immanent object (2) the act or noesis (3) the noema as the result and correlate of this act. Another way to express the relation is to say that whenever we perceive something, upon closer scrutiny we perceive “something as something.” The first something is the immanent object, the second one is the noema—and both are connected by means of the conscious act, the noesis.

The formula “something appears as something” is also called “intentionality.” Intentionality is an umbrella term for a wide variety of conscious acts. In fact, our experience of the world *as such* rests on countless *conscious acts*. These noetic acts also have a result or effect, namely the noema. These acts are carried out by you. Therefore, you can say that *you intend* that red thing on the kitchen table as an apple. But upon closer inspection, it may turn out that it is actually a tomato. This would show that you can be aware of the way *you* intend a noema, though the transcendent object might call for a different way to intend it^17^. This also explains why, when you are *searching* for something, in a sense you already experience what you search—namely the noema as a way to intend that particular thing. Yet you only *find* what you search for when the *thing* that you intend also *presents itself* in an actual perception (cf. Brandl, 2005, pp. 170–71).

When something appears and you intend it as something, the relation is *not one of causation*, but of “motivation,” as Husserl (1983, p. 107, 1977, pp. 107–108) calls it. That red thing *motivates you* to intend it as an apple; it does not cause you to intend it as an apple. In everyday life, most motivations and their respective intentions occur passively (see Husserl, 1966). Yet, via epoché, you can become aware of these acts, and once you are aware of them, you are free to try out different intentions, e.g., “cherry,” “pear,” and so on. If you do, most of the time the intention will not be appropriate, so the freedom is not to see the world as you please. The word motivation is employed because there are not just two factors involved, like cause and effect in mechanical causality, but also a subject. The immanent object thus motivates *you* to intend it as this rather than that noema, but you are free to try out a different one. This freedom underlying our experience of the world, the related possibility to err and the involvement of a subject, are the reason why it is appropriate to speak about *your intention* to see it this rather than that way.

Noticing the noesis means to become aware of a *constituting activity* that constantly underlies the experience of the world as we know it. This shows that phenomenology is not armchair reflection, but is the study of actual mental processes. The goal of phenomenology is to discover and describe consciousness by means of studying the essential conscious elements, acts, structures, and their interrelation. In order to further understand Husserl's method, the acts of imagining and perceiving must now be contrasted.

### Imagination vs. perception

Imagine an elephant in a whirlpool. Now reflect: What just happened in your consciousness? While you imagined this scenario, you probably experienced something grayish for a moment. Notice that this grayish experience occurred *in addition* to your ongoing sensory impressions. Also, you were aware that this grayish experience was not something you perceived with your eyes. Instead it was something you experienced because you were imagining an elephant. During that attempt, your ongoing sensory impressions formed a kind of background to your imagination of the elephant, which was in the foreground of your awareness. The image of the elephant was also probably more unstable compared to the sensations underlying your current perceptions, e.g., those of the words in this text.

These differences with regard to ongoing sensations were the reason Husserl used a different word to name the experiences occurring in imagination. He called them “phantasmata” (singular: “phantasma”). Phantasmata make up the immanent objects in acts of imagining. In an act of perception, however, you experience a sensation and try, based on this sensation, to perceive the correlating object. This means that the sensation has the upper hand and you try to intend the adequate intentional object. This is usually reversed in acts of imagining. When you wish to imagine something, you know *at the outset which intentional object* you wish to imagine. Your efforts are then geared toward experiencing a phantasma that is a suitable basis for imagining the intentional object. Usually it takes quite some practice to experience a stable phantasma, as becomes evident when looking at Buddhist meditation techniques (see Wallace, 1999).

In contrast to imagination, Husserl (1970, p. 105) saw perception with its underlying sensations as “the primal mode of intuition.” Nonetheless, he made imagination the foundation for his phenomenological methodology. In order to understand why, Husserl's concept of categorial intuition together with eidetic variation must be introduced.

### Categorial intuition and eidetic variation: introspective explorations

In the sixth of his *Logical Investigations*, Husserl (2001b, pp. 181–334) was interested in how we assess the truth of a proposition in light of the perception it describes. A proposition always entails categories, like “is” or “causes.” For Husserl, categories are not a fixed number of concepts, but a general term for *conceptual relations*. Conceptual relations cannot have a *direct* (i.e., one to one) correlation among individual sensations (or phantasmata). Husserl claimed, however, that if the *individual relata* connected by means of the conceptual relation are *presented intuitively*, the *meaning* of the category can achieve an *intuitive fulfillment* as well. He called this “categorial intuition.”

Husserl (2001b, pp. 292–293) thereby discovered the curious fact that the meaning of a category is *equally well fulfilled* if its relata are intuitively given as sensations *or* phantasmata. In other words: The differences between perception and imagination play no role in intuitive fulfillment of a category as such. This applies not only to relational concepts, but also to unitary ones like “thing”^18^. In order to have the concept “thing” fulfilled by intuition, a phantasma serves just as well as an actual perception. This discovery was the starting point for the phenomenological methodology named “eidetic variation”^19^.

You may take a concept like “thing” and start imagining different possible experiences of it. Husserl (1973b, p. 341) observed that it “then becomes evident that a unity runs through this multiplicity of successive figures, that in such free variations of an original image, e.g., of a thing, an *invariant* is necessarily retained as the *necessary general form*, without which an object such as this thing, as an example of its kind, would not be thinkable at all.” He explained: “This general essence is the *eidos*, the *idea* in the Platonic sense, but apprehended in its purity and free from all metaphysical interpretations, therefore taken exactly as it is given to us immediately and intuitively in the vision of the idea which arises in this way” (Husserl, 1973b, p. 341). The name “eidetic variation” expresses this way to intuit an *eidos* by means of producing lots of possible *variants* in order to achieve intuitive awareness of the underlying necessary general form.

Eidetic variation is a compromise between Hume and Kant. Hume required grounding claims about conceptual relations like causality *in corresponding intuitions*. Kant, however, *rejected* grounding them on inductions based on *perceptions*, as this could never prove their necessity.

Husserl's solution is to ground claims about a priori laws of consciousness not in perceptual intuition (sensation), but in free variations of imaginings (phantasmata). Categorial intuition plays a key role with regard to Kant's claim that there are synthetic a priori judgments like causality, which need a non-conceptual carrier. In eidetic variation, phantasmata form the intuitive basis of such categorial a priori judgments (see Jansen, 2005, p. 127). The essential law that a physical thing cannot be visually presented from all sides and distances is an example of a categorial judgment that achieves fulfillment by means of eidetic variation. According to Husserl, whatever is *essential* (necessary) about a concept (eidos) becomes *intuitively evident* in its eidetic variation. Thus, eidetic variation is not armchair speculation, as it bases its theoretical claims on actual intuition.

Husserl (1983, p. 11) also maintains that “*[p]ositing of and, to begin with, intuitive seizing upon, essences implies not the slightest positing of any individual factual existence; pure eidetic truths contain not the slightest assertion about matters of fact.”* In other words: Results of eidetic variation are not a posteriori judgments dependent on actual perception. Instead, they are judgments about a priori structures of possible experiences. Thus, the freedom of variation overcomes the confines of inductive methodology, which is dependent on facts presented to us via sensation. While imagination thus “*makes up the vital element of phenomenology*” (Husserl, 1983, p. 160), varying phantasmata is nonetheless not the goal of eidetic variation, but its means to achieve intuition of essential laws.

Eidetic variation is thus the answer to questions (4) and (5). Grounding eidetic variation in imagination rather than actual perception, Husserl strives to overcome the shortcomings of empirical induction. Also, an essential law (*Wesensgesetz*), e.g., that no physical thing can be seen from all sides and distances simultaneously, is not something that is peculiar to an individual's consciousness. It is not even peculiar to a culture—it holds in China just as well as in Chile. The individual variants that different individuals run through in imagination in order to intuit an eidetic structure do in fact differ. But the law itself abstracts from these peculiarities: Husserl (1973b, p. 341) states that during eidetic variation “what differentiates the variants remains indifferent to us” ^20^

Just like when conducting mathematical calculations, erroneous judgments while performing eidetic variation are possible. This is where intersubjectivity enters as a welcome and helpful corrective. Precisely because eidetic variation abstracts from the observer-dependent peculiarities, others may confirm or disconfirm my descriptions, as they experience the same essential structures. Thus, though the method is quite different, there is a way in the phenomenological description of consciousness, just like in science, to achieve objective, in the sense of observer-independent, descriptions^21^.

Here it is appropriate to discuss the last missing feature of introspection Schwitzgebel mentions. It is called the “*first-person condition*: Introspection is a process that generates, or is aimed at generating, knowledge, judgments, or beliefs about *one's own mind only* and no one else's, at least not directly” (Schwitzgebel, 2016). As Husserl strives to experience the *general* laws of consciousness, he was not interested in describing peculiar or idiosyncratic aspects of *his individual* mind. Instead he wanted to experience himself those laws and structures that are in effect in the minds of others as well. However, the experience of these laws' generality in eidetic variation is not such that one experiences them as effective in one's own *and in other minds*. One experiences them in one's own mind only. So there is no direct experience of other minds involved. Therefore, if the first-person condition refers to experiences idiosyncratic to one's own mind, Husserl's phenomenology focuses on experiences (essential structures) that do not have this feature. If it means that the general laws are experienced in one's own mind only and not in other minds, Husserl's method has this feature.

## Husserl's distinction between phenomenology and introspection

Phenomenology's *directly intuiting essential* laws governing consciousness was the reason why Husserl saw it as distinct from psychology's inner observation. In his understanding, psychology treats conscious phenomena as *singular empirical facts* and tries to *induce generalities* from recurring observations (see Husserl, 1983, p. xx; Cai, 2013, p. 15). Such a treatment of conscious phenomena is possible, of course. Yet it would be indeed different from phenomenology, for if one limits the meaning of the word “introspection” to this proceeding, phenomenology would be something else. Still, both this narrow meaning of introspection and eidetic variation look in the same direction and in fact strive for something similar.

They strive for something similar in that an empirical inductive proceeding is ultimately not interested in *individual* empirical facts. Instead, it collects them as a means to induce *generalities*. It must thereby rule out certain empirical observations, although they are actual empirical facts, as mere noise in order to achieve an understanding of the general laws governing these observations. While this access to the generalities is indirect, inductive, and statistical, it is nonetheless interested in something quite akin to essential structures.

Both proceedings look in the same direction, in that phenomenology does not speculate about essential structures governing consciousness; it *intuits them as they are given in consciousness*. Eidetic variation's intuiting is not only intricately interwoven with possibly idiosyncratic experiences—namely the phantasmata it varies—it depends on them as its foundation. Even though, building on this, it then singles out something that empirical induction ignores or does not “see,” it is nonetheless *partly* a way of looking at one's own consciousness in the very same direction as introspection in the narrow sense. Furthermore, the intuition of essences is itself directed at something given intuitively in consciousness. Thus, if introspection means to study one's consciousness *by means of actually observing it*, eidetic variation *introspects* both phantasmata and essences.

## Critical discussion of husserl's method

Now that Husserl's method has been outlined along with some of its achievements and potential, it is crucial for an interdisciplinary dialogue on introspection to at least sketch some of its problems and weaknesses.

First of all, most of Husserl's descriptions concern seeing. While he did provide some descriptions of hearing and touching (see Husserl, 1973a, 1991a,b), those of smell and taste are scarce. Also, the areas of willing and feeling are comparatively underdeveloped. Furthermore, how we achieve consciousness of what others think, feel and want remains problematic. Husserl's solutions, e.g., that I project a modulation of my ego into the other based on associative pairing of his and my body (see Husserl, 1960, pp. 89–150), often seem speculative rather than truthful to experience. The aspects mentioned therefore need an extension, possibly even a modification of the method.

A serious critique by Depraz et al. (2003, p. 70) is that Husserl and many phenomenologists following him “never bother to ask themselves how they're able to write as phenomenologists.” Depraz et al. (2003, p. 65) correctly “insist that intuition, on the whole, can be accomplished without expression.” This is a critique of how one accurately conveys an experience in the medium of language, which is a process comparable to a translation. To be sure, for descriptions, Husserl (1983, p. 151) requires that “the concepts used actually conform faithfully to what is given,” but he does not go into detail about how to achieve this methodologically. Therefore Depraz et al. (2003, p. 70) are right that “expressive fulfillment remains a blind spot in phenomenological analysis,” and that “Husserl barely treated it.”

What complicates matters is that phenomenologists like Merleau-Ponty (2002, p. 54) claimed “a significance of the percept which has no equivalent in the universe of the understanding.” For if there is an unbridgeable gap between *experiential meaning* and *descriptive concepts as the result of reflecting on experience*, a phenomenological description would be doomed to failure. Related is the strong contrast in phenomenology between life-worldly pre-reflective meaning and objective concepts as the goal of scientific descriptions. This issue is a complex one and cannot be resolved here. What is required is a detailed understanding of the differences and relations between, on the one hand, experience, pre-reflective meaning, reflective concepts, and words as elements of a language, and, on the other hand, the acts of perceiving, thinking, judging, and speaking.

Husserl's subjectivization of constitution is also questionable. An example: If I walk down a street, my walking is subject to the law of gravity. But even though walking is *my activity*, it would be incorrect to assume that gravity is *subjective*. Yet Husserl seems to assume that the transcendental subjectivity is not only involved in constitution, but that the constituting activity is subjective. He probably assumes this, as he also assumes an a priori innate to subjectivity. It could be, however, that the transcendental constitution is *in accordance* with a priori laws without the subject having to be *the source* of these laws. The subject could *partake* in these laws whenever active, like our walking partakes in gravity.

The inherent development and dynamics of consciousness pose further problems. For instance, Husserl (1960, p. 74) described the experience of an infant as *eidetically different* from that of an adult. If, however, the eidetic structure of consciousness itself is subject to change, whatever we find for adults is not necessarily transferable to other stages of conscious human life. This need not endanger generalizability within the conscious life of adults. But a newborn cannot practice eidetic variation, much less give an adequate report of what her consciousness is like. Accordingly, Husserl's attempts to explain the origin of consciousness are problematic.^22^

Finally, Husserl's belief that concepts and eidei are in need of a sensory foundation to be experienced as meaningful is problematic. Crowell (2016, p. 193) notices that “to recognize […] the fulfillment relation […] is not yet to provide a phenomenology of thinking.” Eidetic variation is no doubt useful to determine whether a sensory experience *can* correspond to an ideal meaning. In this sense, as Jansen (2005, p. 127) puts it, it can serve “as an illustrative *model* for experience.” Yet, as the ideal meaning functions also as the *criterion* to determine the *degree of sensory fulfillment*, Husserl's phenomenology presupposes, but does not explore, the experience of thoughts themselves.^23^

## Results

The discussion of phenomenology in relation to the six introspective features that Schwitzgebel mentions showed how phenomenology can not only be seen as a kind of introspection, but also as a quite sophisticated method for practicing it. The principle of all principles ensures that claims have a foundation in actual intuition (*spectare*) and thereby prevents arbitrary speculations about unobserved entities. The epoché shifts awareness away from the transcendent world and aids paying attention to consciousness as such.

The remaining two methodological steps then help to put results on a secure basis. The reduction makes sure that the meanings employed in the description are in full concordance with the actual experience. This allows the notice and elimination of false prejudices. The eidetic variation further helps to test claims about necessary structures without being dependent on actual perception. The freedom of this variation helps overcome the limitations of empirical induction. Its results are generalizable, as it makes only indirect use of the possibly idiosyncratic phantasmata. Lastly, intersubjective testing of the results is as important in phenomenology as it is in science.

One of Husserl's greatest achievements was to disprove the Kantian prejudice that the world is only our representation. As long as one is blinded by this prejudice, one cannot even clearly distinguish between *intro*-spection and *extro*-spection. Husserl, however, showed how we can be *conscious of* something which is itself not of consciousness. Therefore, we can be conscious not only of the psychological, but also of the physical and ideal planes and strive for a clearer understanding of these different layers and their relations. Yet phenomenology only studies the layers of consciousness and leaves out the ones transcendent to consciousness. Identifying and understanding all the layers as well as their interplay is thus a task for which natural science is as important as phenomenology and psychology.

## Author contributions

The author confirms being the sole contributor of this work and approved it for publication.

### Conflict of interest statement

The author declares that the research was conducted in the absence of any commercial or financial relationships that could be construed as a potential conflict of interest.

## Footnotes

^1^Depraz et al. (2003, p. 7) portray their approach as “a re-awakening of introspective psychology.” However, they criticize Husserl's “intellectualist tendency” (2003, p. 161) and choose an “entirely pragmatic” (2003, p. 1) approach. Instead of combining it with other approaches, the following article seeks to show how Husserl's method—in and of itself—is a kind of introspection.

^2^Naturalistic painters are a notable exception to this: They need to imitate the way the world appears in consciousness in order for us to be able to see what they want to depict.

^3^While Husserl (1983, p. 218) rejects the term “mental”, he still wishes to explore the way we are *conscious of* the *mind-external* world, among other levels of consciousness, thereby fulfilling Schwitzgebel's definition.

^4^Zahavi (2015, p. 185) compares reflecting on something one is aware of in a pre-reflective manner “to the relation between marginal and focal consciousness. In both cases, the transition from one to the other can be understood in terms of an *attentional modification*.” I leave open here, however, whether awareness of something genuinely new could arise as well.

^5^Schwitzgebel (2016) portrays Husserl's phenomenology as a containment approach and counts it among the “ways of obtaining self-knowledge [that] all violate the detection condition on introspection.” He writes: “Husserl (1913/1982) offers an early phenomenal containment approach, arguing that we can at any time put our “cogitation”—our conscious experiences—consciously before us through a kind of mental glancing, with the self-perception that arises containing as a part the conscious experience toward which it is directed, and incapable of existing without it.” I agree with Schwitzgebel's characterization, but I would hold that the epoché is the “mental glancing” he mentions, and it is a necessary means *to detect* the conscious life in the manner described.

^6^Materialistic approaches like Dennett's (1992, p. 41) claim that “[s]omehow the brain must be the mind” and thus seek to eliminate all reality of conscious experience as such. To some extent, this is even in line with Husserl (1983, p. 111), who stressed that “a veritable abyss yawns between consciousness and reality.” Thus, for Husserl, it would be wrong to seek consciousness as something real in the materialist sense, as the being of consciousness is on an entirely different level. Dennett instead proposes what he calls “heterophenomenology”: He lets subjects report on their conscious experience and correlates these supposedly objective reports to brain events. This is reminiscent of the “thinking aloud” turn in the history of psychology that, instead of developing a method of introspection, treated verbal reports of subjects on their conscious experience as objective data. Vermersch (1999, p. 27) criticizes these approaches because they “forget that, in order to produce these verbalizations, the subject has to have access to something even to be in a position to describe his mental acts.” Similarly, Cerbone (2003, p. 134) criticizes Dennett's heterophenomenology because it “helps itself to scientific data of all kinds […] whose possibility as data is left unexplained.”

^7^Ni (1998) discusses the meanings, possibilities, and limitations of reflection within phenomenology.

^8^A mirror or video only seem to change this, as they provide not David's appearance, but a *depiction of* David.

^9^Touch may of course provide impressions of different sides of an object simultaneously, but it does not present colors. Therefore, invoking different senses only emphasizes that we cannot simultaneously experience all possible sensations. Husserl (1973a, p. 156) called it a most difficult question how the different impressions are unified as qualities of the same object.

^10^Husserl (1983, p. 197) called the implied idea of a complete sensory givenness an “*idea in the Kantian sense*.” Husserl (1983, p. 197) argued that this idea, as such, can reach “an absolutely indubitable givenness.”

^11^Kelly (2014, p. 560) mentions a third meaning of “transcendent,” namely the sphere of god and other theological or metaphysical entities. As Husserl does not often ponder this kind of transcendence, I leave it out.

^12^The noema, as an object of consciousness, correlates with a conscious act. Husserl calls this act the “noesis.” The noesis is introduced in more detail below.

^13^To my knowledge, Husserl did not call the noema a “transcendental object.” Yet if one wishes to understand the relation to Kant or why Husserl spoke of a *transcendental* phenomenology, this classification helps. Zahavi's (2015, p. 187) explanation of the ontological distinction takes a similar direction.

^14^An important difference being Kant's claim that knowledge of the things in themselves is entirely impossible.

^15^Even though this is so, one can of course err as soon as one *apperceives* the object *as* this or that.

^16^Husserl uses “epoché” and “reduction” interchangeably (see Depraz, 1999, p. 100; Staiti, 2009b, p. 86). Consequently, the question of whether and where to draw a distinction between the two is often disputed. The distinction made here is akin to the one Zahavi (2003, p. 46) suggests.

^17^In philosophy of mind, the term “intentionality” has a different meaning, which Kriegel (2011, p. 80) defines as follows: The “source of intentionality is a certain natural relation that obtains between internal states of the brain and external states of the world.” It is thus important not to confuse the two meanings (see Breyer and Gutland, 2016, p. 6).

^18^Husserl (1969, p. 248) explicitly included syntactic objects (categories) within the meaning of “eidos.”

^19^Husserl's proceeding from categorial intuition toward eidetic variation is not without problems. One of the most pressing ones is the question how intentions of essences/categories are given in contrast to their fulfilling intuition, as well as their interrelation (see Lohmar, 1990). Discussing these issues, however (let alone solving them), is beyond the scope of this paper (but see Gutland, forthcoming).

^20^Ströker (1983, p. 10) distinguishes between (a) fact and eidos (*Wesen*) and (b) between being (*Seiendem*) and its phenomenon. In the terminology used here, eidetic variation is the means of acquiring knowledge about eidei rather than empirical facts, while epoché is the means to experience phenomena rather than being.

^21^While an important corrective, one must not overemphasize or misunderstand the relevance of intersubjectivity: In phenomenology, essential structures are not necessarily identical to what the *majority* of the intersubjective phenomenological community claims them to be. As Husserl (2008, p. 658, my translation and emphasis) stressed, “The normal as an optimum *distinguishes itself in experience* so that even a single person could become the norm of the experiential truth.” Husserl (2008, pp. 658, 721) illustrates this: If most people were born blind, this sheer majority would be no reason to reject the possibility of the minority's experience of color. Thus, the criterion for phenomenologically accurate descriptions remains the self-givenness of the intended experience. Like in science, if many people employ the same kind of experimental setting and thereby achieve the same result, this can foster, but never substitute objective descriptions.

^22^Husserl's attempts to hypostatize instincts within the ego (see Husserl, 2008, pp. 474–77) or to “transcendentalize” Hume's law of association (see Husserl, 1968, p. 286) are violations of the principle of all principles and thus speculative.

^23^I (see Gutland, forthcoming) have argued that the experience of thoughts is *non-intentional*: There is no given *something* which needs to be intended *as something else*. Instead, thought experience is *inherently meaningful*, rendering additional meaning-bestowing acts superfluous. This is in line with findings of several authors debating a cognitive phenomenology (see Levine, 2011, p. 109; Siewert, 2011, p. 262; Pitt, 2011, p. 141).
